# Systemic Absorption of Oral and Rectal Vancomycin in a Critically Ill Patient: A Case Report

**DOI:** 10.7759/cureus.76008

**Published:** 2024-12-19

**Authors:** Rastko Rakočević, Keith Guevarra

**Affiliations:** 1 Pulmonary and Critical Care Medicine, Los Angeles General Medical Center, Los Angeles, USA; 2 Pulmonary and Critical Care Medicine, Rutgers New Jersey Medical School University Hospital, Newark, USA

**Keywords:** c diff, clostridioides difficile infection, clostridium difficile, colitis, critical care, infectious disease, pulmonary critical care, sepsis, vancomycin, vancomycin absorption

## Abstract

Enteral administration of vancomycin is the standard treatment for *Clostridioides difficile *(*Clostridium difficile*) colitis and is presumed to have no systemic absorption. In critically ill patients, however, especially with multi-organ failure, enteral absorption of vancomycin is unpredictable and can cause severe toxicity if it remains unrecognized. We therefore report a case of systemic absorption of enteric vancomycin in a patient with severe *C. difficile *colitis.

## Introduction

Vancomycin is a commonly used antimicrobial agent, whose toxicities can be severe and are thought to be related to its elevated serum levels [1,2]. Enteral administration of vancomycin (VAN) is the standard treatment for *Clostridioides difficile* colitis (*C. diff* colitis) and is presumed to have no significant systemic absorption [3]. While parenteral use of VAN requires frequent therapeutic drug monitoring due to its narrow therapeutic index, this is not a clinical practice for enterally administered VAN. We report a case of systemic absorption of enteric VAN in a patient with severe *C. diff* colitis, treated with oral (PO) and rectal VAN.

## Case presentation

**Figure 1:**
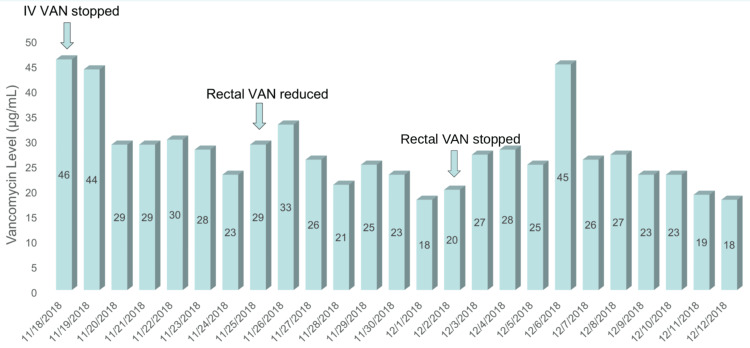
Daily vancomycin serum levels initially and after reducing and stopping rectal vancomycin IV: intravenous; VAN: vancomycin

A 59-year-old woman with a history of antineutrophil cytoplasmic antibodies (ANCA) vasculitis (on immunosuppressants), fungal infections (on prophylactic voriconazole), and hypothyroidism (on levothyroxine) was hospitalized for bilateral hip pain and fever. Hip joint washout and blood cultures showed *Salmonella *sp. (treated with ceftriaxone). The hospital course included acute hypoxic respiratory failure, septic shock, disseminated intravascular coagulation (DIC), and acute renal failure. She received a short course of intravenous (IV) VAN and meropenem empirically and was placed on continuous renal replacement therapy. She subsequently developed severe *C. diff* colitis and was treated with VAN 500 mg every six hours (q6h) PO, VAN 500 mg q6h, rectal and IV metronidazole. IV VAN was discontinued the same day and VAN random blood level (VRL) at the time was 46 µg/mL. VRL was obtained daily at 5 AM, and for the next two days, levels were 44 µg/mL and 28 µg/mL respectively. For the next 14 days after VAN IV was discontinued, VRL remained at a steady mean level (x̅) of 25.14 µg/mL, SD ±4.18 even after the reduction of rectal VAN dose to 250 mg q6h. The patient was switched to intermittent HD and rectal VAN was discontinued. In the following 10 days, VRL remained elevated at x̅= 25 µg/mL, SD ±4.61 (Figure 1).

Albumin and total protein levels were x̅= 2.16 g/dL, SD ±0.33, and x̅= 4.25 g/dL, SD ±0.47, respectively.

The hospital course had multiple other complications which included *Staphylococcus epidermidis* mitral valve endocarditis (treated with daptomycin), subacute infarction on MRI brain, quadriplegia with areflexia with no obvious etiology (inconclusive electromyography (EMG)/neurogenic stress cardiomyopathy (NSC), negative lumbar puncture (LP)), worsening hearing impairment and right brachial and left common femoral deep vein thrombosis (DVT). She was subsequently stabilized and transferred to a long-term acute facility.

## Discussion

*C. diff* colitis is inflammation of the colon caused by disruption of physiologic bowel flora, most commonly due to antibiotic use. First described in 1974 as a rare phenomenon associated with clindamycin use, *C. diff* colitis has now become a more toxic, and more refractory disease reserved no longer only for hospital setting but also acquired in the community [4], with clinical manifestations ranging from self-limited diarrhea to fulminant pseudomembranous colitis.

Critical care patients are a specific subgroup of patients with *C. diff *colitis because these cases are commonly severe and require both IV and enteral treatment, and because offending antibiotic agents are often challenging to discontinue due to infections with multiple drug resistance bacteria [5]. Systemic absorption of enterally administered VAN is rare and routine serum drug monitoring is not recommended [3]. Current Infectious Diseases Society of America (IDSA) practice guidelines for CDI in adults suggest it might be appropriate to monitor serum VAN concentrations in patients with renal failure, disrupted intestinal epithelial integrity, and prolong exposures of high doses of both PO VAN (2 g/day) and rectal enemas (500 mg doses).

In a study performed on 57 patients with severe *C. diff *colitis, 98% did not have a detectable VRL [6]. However, rare case reports describe VRL elevation with enteral VAN administration, causing flaccid paralysis, encephalopathy, and ototoxicity, usually in patients with renal failure and severe *C. diff* colitis, similar to our patient [5-11]. The proposed mechanism is that small amounts of enterally absorbed VAN due to inflammation in severe* C. diff* colitis, 'lingers' longer and accumulates in patients with impaired renal function. Since vancomycin can also cause renal dysfunction on its own, it is sometimes hard to tell which one came first: renal impairment causing elevated VRL or elevation of serum VAN causing nephrotoxicity. No matter the cause, in reported cases, decreasing VAN dose and/or starting hemodialysis caused swift resolution of elevated VRL, in a matter of few days [1,2,7-11]. This was not the case in our patient, who had significant, toxic levels of VAN in blood for >2 weeks [12].

Other patients with elevated VAN levels in the blood had gastroenterological (GI) complications or surgical interventions (small bowel infarction, hemicolectomy, severe pseudomembranous colitis [10,11,13-15]). Since our patient did not have a colonoscopy or endoscopy, we can not say for sure if she had anatomical bowel disruption. One study showed that 15 out of 85 (17.6%) adults receiving oral vancomycin for severe *C. diff* colitis had VRL ≥2.5 mg/L [16]. This single-centered study identified the presence of GI pathology, ICU admission, use of VAN retention enemas, and having a creatinine clearance ≤ 50 ml/minute or undergoing hemodialysis or continuous renal replacement therapy as risk factors for systemic vancomycin absorption. Notably, in the same study, four out of nine patients who received vancomycin retention enemas had serum vancomycin concentrations of >2.5 mg/L. It is important to emphasize that in the majority of all reviewed cases in the literature [1,2,3-9,11,13-16], VRL concentration was <2 mg/L, and only one had reported VRL concentrations of >15 mg/L for >2 consecutive days, which resolved completely a few days after stopping enteral VAN [10]. While levels < 15 mg/L with prolonged exposure (>10 days) may be associated with the development of resistant strains of *Staphylococcus aureus* including VAN-intermediate *S. aureus* (VISA) and VAN-resistant *S. aureus* (VRSA) [17-19], systemic VAN concentrations of >15 mg/L are associated with ototoxicity, nephrotoxicity and, rarely neurotoxicity [11, 20].

Based on this, our patient had all the risk factors for increased absorption of enteral VAN. However, despite the continuation of oral VAN, it remains unclear why the VAN levels remained >15 mg/L for such a long time, even after stopping rectal VAN and despite regular hemodialysis. Besides the already discussed worsening in renal function, our patient also developed worsening hearing impairment and flaccid paralysis. This was thought to be due to prolonged exposure to toxic levels of serum VAN causing severe toxicity, not otherwise explained by MRI, EMG/NSC, or LP.

## Conclusions

This report described the case of a critically ill patient with sepsis and renal failure who developed severe *C. diff* colitis and whose treatment with enteral vancomycin was complicated with significant systemic absorption and associated toxicities. Larger prospective studies are necessary to demonstrate the extent of enteral vancomycin absorption and its consequences in critically ill patients. Until then, due to the possibility of developing severe VAN toxicity, we propose that besides obtaining periodic VRL in all patients with discussed risk factors, physicians should check VRL in all critically ill patients treated with enteral VAN for severe *C.diff* colitis who are demonstrating any sign of possible VAN toxicity.
